# Moderate-High Blood Eosinophilia Is Associated with Increased Hospitalization and Other Asthma Comorbidities

**DOI:** 10.3390/biom14010126

**Published:** 2024-01-18

**Authors:** Sara Naharro-González, Clara Lorente-Sorolla, José Manuel Rodrigo-Muñoz, Marcela Valverde-Monge, Erwin Javier Pinillos-Robles, Diana Betancor, Mar Fernández-Nieto, Diana Sánchez-Mellado, Marta Gil-Martínez, Jessica Mireya Santillán-Coello, José Miguel Villacampa-Aubá, Ignacio Mahillo-Fernandez, Antonio Herrero-González, Alejandro Perez-González, María Jesús Rodríguez-Nieto, Victoria del Pozo

**Affiliations:** 1 Immunoallergy Laboratory, Immunology Department, Fundación Jimenez Díaz Health Research Institute (IIS-FJD, UAM), 28040 Madrid, Spain; sara.naharrog@quironsalud.es (S.N.-G.); clara.lorente@quironsalud.es (C.L.-S.); jose.rodrigom@quironsalud.es (J.M.R.-M.); marta.gilm@quironsalud.es (M.G.-M.); 2 CIBER of Respiratory Diseases (CIBERES), Instituto de Salud Carlos III (ISCIII), 28029 Madrid, Spain; marcela.valverde@quironsalud.es (M.V.-M.); diana.betancor@quironsalud.es (D.B.); 3 Allergy Department, Fundación Jiménez Díaz University Hospital, 28040 Madrid, Spain; mmfernandez@fjd.es; 4 Pulmonology Unit, Fundación Jiménez Díaz University Hospital, 28040 Madrid, Spain; erwin.pinillos@quironsalud.es (E.J.P.-R.); diana.sanchez@quironsalud.es (D.S.-M.); mjrodriguezn@fjd.es (M.J.R.-N.); 5 ENT and Cervicofacial Surgery Department, Fundación Jiménez Díaz University Hospital, 28040 Madrid, Spain; jessica.santillan@quironsalud.es (J.M.S.-C.); jmvillacampa@fjd.es (J.M.V.-A.); 6 Department of Epidemiology, Fundación Jiménez Díaz University Hospital, 28040 Madrid, Spain; imahillo@fjd.es; 7 Data Analysis Department, I Fundación Jimenez Díaz Health Research Institute (IIS-FJD, UAM), Fundación Jiménez Díaz University Hospital, 28040 Madrid, Spain; aherrero@quironsalud.es (A.H.-G.); alejandro.perezgnz@quironsalud.es (A.P.-G.); 8 Villalba General University Hospital, 28400 Madrid, Spain; 9 Department of Medicine, Autonomous University of Madrid, 28049 Madrid, Spain

**Keywords:** blood eosinophils, diagnosis, biomarker, asthma, CRSwNP, COPD, hospitalization

## Abstract

(1) Background: Eosinophilia has traditionally been linked to eosinophilic asthma, for which it is the gold-standard prognostic biomarker. However, the association between eosinophilia and the presence of other diseases and comorbidities is yet unclear. (2) Methods: For this retrospective study, we reviewed the electronic medical records of 49,909 subjects with blood eosinophilia to gather data on the presence of asthma, COPD, sleep apnea, tuberculosis, dyslipidemia, hypertension, and other cardiovascular diseases and severe CRSwNP among these subjects. Demographic features including age, sex, and smoking habits were collected, as well as the number of hospitalizations and emergency department visits. T-tests, ANOVA, Fisher test, and logistic regression models were used. (3) Results: For all age groups studied, eosinophilia was significantly more prevalent among asthmatic subjects than nonasthmatics, especially in patients also presenting CRSwNP, hypertension, and dyslipidemia. The likelihood of developing asthma, COPD, and CRSwNP, and hospitalization, was increased when BEC was above 600 eosinophils/μL. The association between asthma, CRSwNP, and BEC was corroborated by multiple logistic regressions models. (4) Conclusions: We demonstrated the association of having over 600 blood eosinophils/μL with a higher number of hospitalizations and comorbidities (CRSwNP and COPD), which proves that BEC is a highly useful parameter to consider in subjects who present blood eosinophilia.

## 1. Introduction

Eosinophils are terminally differentiated myeloid cells characterized by a segmented, bilobed nucleus and the presence of specific cytoplasmic granules, which play an important role in the immune response to diverse pathogens [1]. Eosinophils have a half-life of 8–18 h in the bloodstream and 48–72 h in tissue, although this period may be longer during inflammatory processes [2]. Primarily located in mucosal tissue, eosinophils are mostly found in the gastrointestinal tract, where the largest population of eosinophils exits (>90% of all eosinophils), with the exception of the esophagus, where there should not be eosinophils [3]. Eosinophils are also located in the spleen, lymph nodes, thymus, mammary glands, and uterus [3].

Eosinophilia is defined as the presence of eosinophil levels above 500 eosinophils/μL in the peripheral bloodstream [4]. Eosinophilia presents different degrees, from mild (500–1500) to moderate (1500–5000) and severe (>5000 eosinophils/μL) [4]. Cases in which the blood eosinophil count (BEC) exceeds 5000 eosinophils/μL, called hypereosinophilia [5], may be associated with diseases like hypereosinophilic syndrome (HES). However, for HES diagnosis, eosinophilia must be accompanied by end-organ damage (e.g., skin, heart, lungs, gastrointestinal tract, nervous system) and symptoms must not be associated with secondary causes [5].

Currently, the clinical approach to eosinophilia is mostly determined by the medical history, as some patients remain asymptomatic, showing no evidence of tissue damage despite an abnormally elevated BEC [6,7]. Most commonly, however, clinical manifestations are severe in cases with both tissue and peripheral eosinophilia as compared to tissue eosinophilia alone [8,9].

When activated, eosinophils contribute to the pathogenesis of various diseases by recruiting other inflammatory cells [3]. Tissue eosinophil infiltration can cause direct damage to tissue due to the cytotoxic effect of the release of granule proteins [3]. One of the diseases where the eosinophil is the main player is severe eosinophilic asthma, an entity in which eosinophil damage causes airway inflammation and remodeling, hallmarks of the pathophysiology of this condition [10]. In many cases, asthma is associated with several comorbidities, such as pulmonary, cardiovascular, and metabolic diseases, thus significantly worsening the clinical condition of patients and increasing healthcare costs [11,12,13]. Thus, the interest in studying comorbidities in asthma is rising and the circumstance of having high blood eosinophil counts with asthma and any comorbidity is of paramount importance for the improvement of clinical practice.

Several studies have reported an association of high BEC with cardiovascular and respiratory diseases, including hypertension [14], asthma exacerbations, chronic rhinosinusitis with nasal polyps (CRSwNP) [15], and chronic obstructive pulmonary disease (COPD) [16]. Recently, eosinophils have also been studied to determine their association with dyslipidemia, one of the main components of metabolic syndrome (MetS), although the results have been inconclusive [17,18,19].

Eosinophilia in the sputum and peripheral blood characterizes the eosinophilic asthma endotype, which is characterized by an inflammatory state of the airways due to infiltration by these immune cells [20]. A high BEC is also related to an increased risk and severity of exacerbation, asthma-related hospital readmissions, and poor disease control [21]. Consequently, BEC is considered a reference biomarker for asthma prognosis, as recommended in recent management guidelines [22,23]. Additionally, BECs above 300 eosinophils/μL can predict the patient response to inhaled corticosteroids (ICS) in asthma [16]. However, certain patients treated with high-dose ICS and long-acting beta-agonist combinations have inadequate symptom control, repeated asthma exacerbations (AEs), or the continued deterioration of lung function [24], referred to as severe eosinophilic asthma (SEA).

Advances in the knowledge of eosinophil biology have led to the development of biological therapies, which are now becoming the new gold-standard treatment for SEA patients and for those with other eosinophilic disorders, who previously had no alternative treatment options. Of notable importance are mepolizumab, reslizumab, and benralizumab, all of which target the IL-5 pathway [25], a cytokine essential to eosinophil activation and survival [4]. Mepolizumab and reslizumab are humanized monoclonal antibodies targeting IL-5, blocking downstream signaling [26]. In contrast, benralizumab, another humanized monoclonal antibody, targets the IL-5 alpha subunit receptor (IL-5Rα), which causes eosinophil depletion by antibody-dependent cellular cytotoxicity (ADCC) [26], likely resulting in a greater reduction in tissue-dwelling eosinophils than mepolizumab and reslizumab [27]. These biologics have been found to decrease the rate of exacerbation [28,29], and mepolizumab and benralizumab can reduce or eliminate the dependence on oral corticosteroids without loss of asthma control [30,31]. Moreover, these therapies have also been approved to treat asthma comorbidities, such as CRSwNP [32] and other eosinophilic disorders, including eosinophilic granulomatosis with polyangiitis (EGPA) and eosinophilic esophagitis (EoE) [28].

The objective of this study was to evaluate and characterize subjects with eosinophilia by quantifying the BEC that is associated with the presence of diverse diseases or conditions. We aimed to gain insights into the prevalence of individuals with eosinophilia in our hospitals, understanding their medical conditions—specifically, the relationship between BEC levels over 300 cells per microliter and the presence of asthma and another comorbidities. Our goal was to explore potential approaches that could enhance the clinical management of patients with blood eosinophilia, deciphering how increasing eosinophil numbers affect asthma and its comorbidities, especially in the context of biologic therapies targeted directly at this cellular population.

## 2. Materials and Methods

### 2.1. Study Design and Data Source

A retrospective study was performed by obtaining data from the electronic medical records (EMR) of subjects admitted to 5 hospitals in Madrid, managed by the firm Quironsalud: Hospital Universitario (HU) Fundación Jiménez Díaz, HU General de Villalba, HU Quironsalud Madrid, HU Infanta Elena, and HU Rey Juan Carlos. The study protocol was approved by the hospitals’ ethics committees and was conducted in accordance with the principles set forth in the Declaration of Helsinki.

The main criteria used for patient selection were the presence of blood eosinophilia (>300 eosinophils/µL) in at least one blood test over the period spanning 2018 to 2021 in patients from ages 14 to 82 years. This cutoff value was selected due to being the cellular biomarker used for the biological treatment of choice (applied for drugs like mepolizumab and benralizumab) for asthma and other eosinophilic diseases like EGPA [28]. We included a total of 53,788 subjects: 49,909 nonasthmatics and 3879 subjects diagnosed with asthma by physicians, based on actual symptomatology, and/or a greater than 12% improvement in FEV_1_ 10 min after the administration of inhaled terbutaline (500 μg; Astrazeneca, Cambridge, UK) or characterized by methacholine airway hyperresponsiveness (PC20 methacholine < 16 mg/mL; Diater, Madrid, Spain), following the Spanish Guidelines for Asthma Management (GEMA 5.3) [22].

### 2.2. Measurement of Covariates and Outcomes

Covariates were extracted from the EMR, including demographic features such as age, sex, and smoking habits. Additionally, clinical parameters and the history of various diseases of interest were extracted from the EMR, either from the diagnostic codes or mined by natural language processing. These diagnoses included respiratory diseases, i.e., asthma (in accordance with the Spanish GEMA guidelines [22]), COPD (as explained in the Spanish COPD Guide (GesEPOC) [33]), sleep apnea (described in the International Consensus Document on Obstructive Sleep Apnea [34]), and tuberculosis (following the World Health Organization guidelines [35]); dyslipidemia, which was evaluated as a component of metabolic disease as stated in the ESC/EAS Guidelines for the management of dyslipidaemia [36]; cardiovascular diseases, i.e., high blood pressure (HBP, as described in ESC/ESH guideline [37]), among others; and, finally, a history of endoscopic sinus surgery (ESS), used in this study as an indicator of the treatment of severe CRSwNP [38,39].

Further information regarding hospitalization and visits to the emergency department was extracted from the EMR.

Regarding the study outcomes, we analyzed the association of BEC and/or asthma with the remaining covariates tested in the model to determine the impact of blood eosinophilia on asthma and other related and nonrelated diseases.

### 2.3. Statistical Analyses

Comparisons between groups were performed using an unpaired, two-tailed T-test for normally distributed samples and the Mann–Whitney U test for samples with a non-normal distribution. ANOVA with Bonferroni post-hoc test was used for comparisons between more than 2 groups of normally distributed samples, and Kruskal–Wallis with Dunn post-hoc test when data were not normally distributed.

Fisher’s exact test was applied to contingency tables to test the null hypothesis of the independence of groups and clinical characteristics (qualitative variables) and the odds ratio (OR) was obtained to determine the association of two sets of variables.

Logistic regression models (univariate and multivariate) were performed to determine the OR and associations between several dependent variables and the independent variable. These included such clinical data and demographic features as age, sex, smoking habits, BEC, hospitalization, emergency department visits, severe CRSwNP, dyslipidemia, HBP, the presence of other cardiovascular diseases and asthma, sleep apnea, COPD, and/or tuberculosis. The independent variable was either the diagnosis of asthma or the BEC value from which eosinophilia was positively associated (OR) with asthma. The logistic regression models included all studied predictive variables to account for any potential disturbance between them.

A *p*-value of less than 0.05 was considered significant. Statistical analyses were performed using GraphPad Prism version 8 (GraphPad Software Inc., San Diego, CA, USA) and the R software (R-4.3.2 for Windows, https://www.r-project.org accessed on 15 June 2023).

### 2.4. Data Sharing Statement

The data that support the findings of this study are available from the corresponding author, VdP, upon reasonable request.

## 3. Results

### 3.1. Study Population

This multicenter study of five participating hospitals in the region of Madrid drew upon data from a study population of 53,788 subjects with a BEC higher than 300 eosinophils/μL. The mean age of the population was 48.51 ± 17.77 years, and 50.55% were female. Although 95.22% of the subjects visited the emergency department during the study period, only 38.71% required hospitalization. All the clinical characteristics analyzed are shown in Table 1.

### 3.2. Asthmatic Patients Have Higher BEC Than Nonasthmatic Subjects in an above 300 BEC/μL Cohort

**Figure 1:**
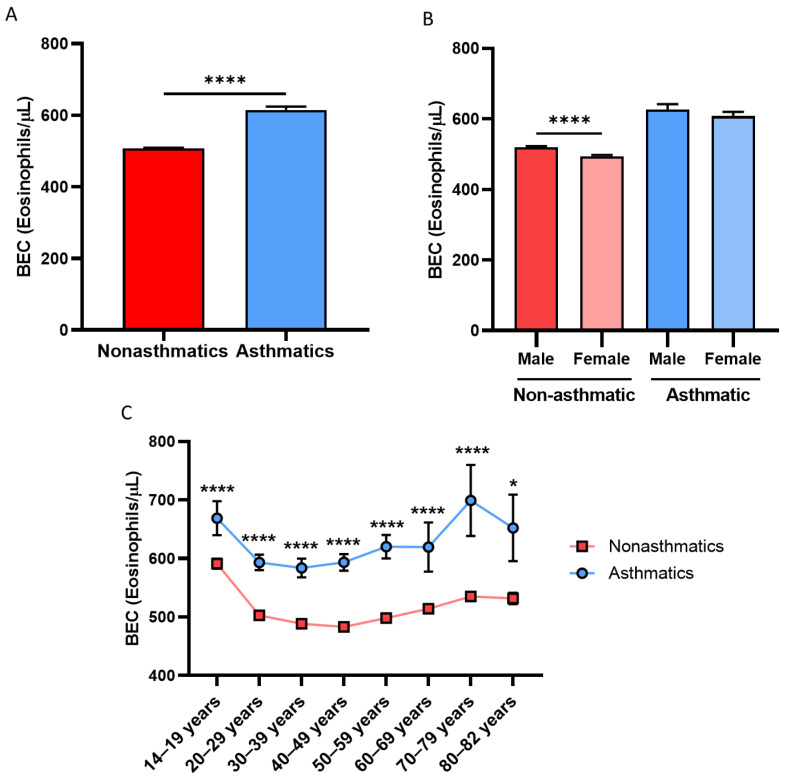
Asthmatic subjects are characterized by higher eosinophilia regardless of sex and age. BEC in (**A**) asthmatic and nonasthmatic subjects, (**B**) according to sex and (**C**) according to age range (14–82 years). * = *p* < 0.05; **** = *p* < 0.0001. BEC: blood eosinophil count.

**Figure 2:**
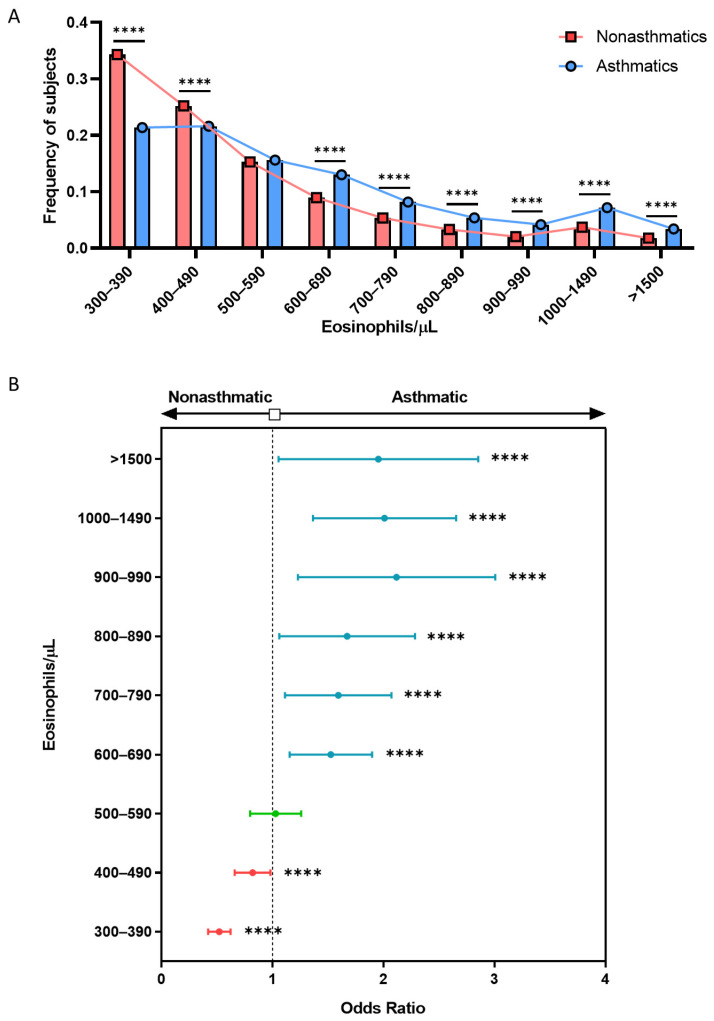
Association of displayed asthma is increased significantly when eosinophilia is higher than 600 eosinophil/μL. (**A**) Frequency of asthmatic and nonasthmatic subjects according to BEC. (**B**) Odds ratio for asthma according to BEC. **** = *p* < 0.0001. BEC: blood eosinophil count; OR: odds ratio. Blue line represents significative positive association, green line means no association and red line represents significative negative association.

The mean value of BEC in the sample population was 510 ± 490 eosinophils/μL. As seen in Table 1 and Figure 1A, the differences observed between asthmatic (610 ± 600 eosinophils/μL) and nonasthmatic subjects (510 ± 480 eosinophils/μL) were significant (*p* < 0.0001). It is noteworthy that, in both groups, the mean BEC was always higher in men, and this difference was statistically significant for the group of nonasthmatic individuals (*p* < 0.0001; Figure 1B). Similarly, the increase in BEC within the asthmatic group remained constant across the spectrum of adult age ranges (Figure 1C). As seen in Figure 2A, a lower percentage of subjects had asthma below the range of 500 to 590 eosinophils/μL; beyond this point, asthmatic patients had significantly higher mean BEC values than nonasthmatic subjects (*p* < 0.0001).

To determine the threshold value above which eosinophilia was a potential asthma biomarker in a cohort with over 300 eosinophils/μL, we calculated the OR for each range of mean BECs, which revealed a marked increase in the association with asthma starting at 600 eosinophils/μL (OR = 1.521; 95% CI, 1.378 to 1.677; *p* < 0.0001; Figure 2B). In both groups, smokers had a significantly lower mean BEC (Figure S1 in the Supplementary Materials), which suggests the importance of the smoking status for BEC.

### 3.3. History of CRSwNP, Hypertension, and Dyslipidemia Is Associated with Eosinophilia in Asthmatic Patients with above 300 BEC/μL

**Figure 3:**
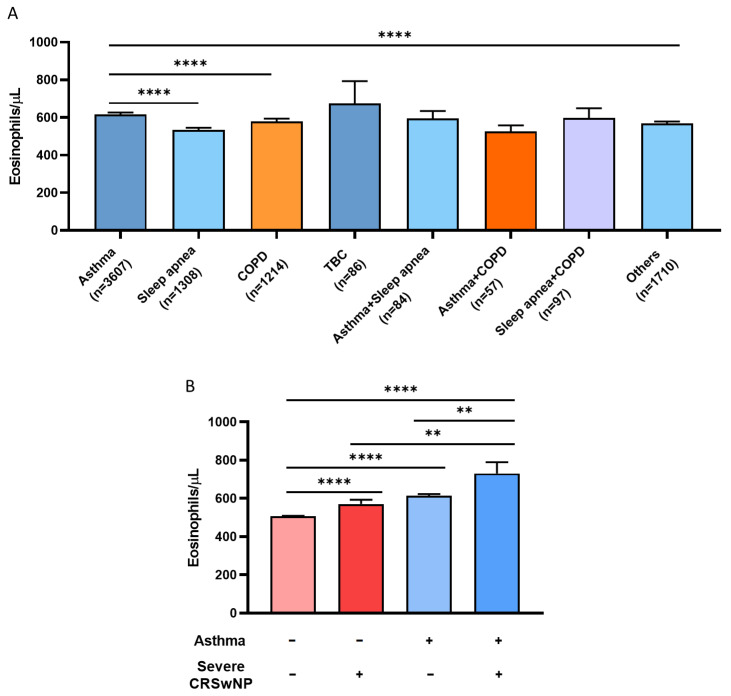
Asthma and CRSwNP are associated with higher eosinophilia than other respiratory diseases. (**A**) BEC in subjects with respiratory pathologies. (**B**) BEC in subjects with CRSwNP, with asthma, with both diseases, or with neither asthma nor CRSwNP (−/−). ** = *p* < 0.005; **** = *p* < 0.0001. BEC: blood eosinophil count; CRSwNP: chronic rhinosinusitis with nasal polyps. + sign represents presence of the disease and − sign represents absence of the disease.

Next, we studied how BEC affected respiratory (pulmonary and nasosinusal), cardiovascular, and metabolic diseases (Figure 3). Tuberculosis was associated with the highest mean BEC (670 ± 1110 eosinophils/μL; Figure 3A). However, the difference with respect to the other respiratory diseases did not reach statistical significance, possibly due to the low prevalence of this illness in the study sample (n = 86). Asthmatics had the second highest mean BEC (620 ± 610 eosinophils/μL), showing statistically significant differences with sleep apnea (530 ± 460eosinophils/μL; *p* < 0.0001), COPD (580 ± 540 eosinophils/μL; *p* < 0.0001), and other conditions (570 ± 460 eosinophils/μL; *p* < 0.0001; Figure 3A).

Regarding nasosinusal diseases, we studied severe CRSwNP by collecting data on the history of ESS. As seen in Figure 3B, asthmatic patients with CRSwNP had the highest degree of eosinophilia (730 ± 450 eosinophils/μL) compared to individuals who, despite having values above 300 BEC/μL, did not have asthma or CRSwNP (510 ± 480 eosinophils/μL; *p* < 0.0001), individuals with CRSwNP (570 ± 330 eosinophils/μL; *p* = 0.002), and asthmatics without CRSwNP (610 ± 600 eosinophils/μL; *p* = 0.001).

The population of subjects who had neither asthma nor CRSwNP comprised a total of 49,687 individuals. Among them, 64.5% did not have any of the diseases evaluated in our study, but 61.6% of them visited the emergency department, and, additionally, 19.3% required hospitalization. The remaining blood eosinophil count (BEC) values were obtained during routine laboratory analytics.

A percentage of 19.2% had dyslipidemia, 22.2% had hypertension, and 9.9% had other cardiovascular diseases. Regarding respiratory pathologies, a total of 2.6% of subjects had apnea, 2.4% had COPD, 0.2% had tuberculosis (TB), and 3.9% had a combination of these pathologies. This left 90.9% of individuals with no reported respiratory conditions.

**Figure 4:**
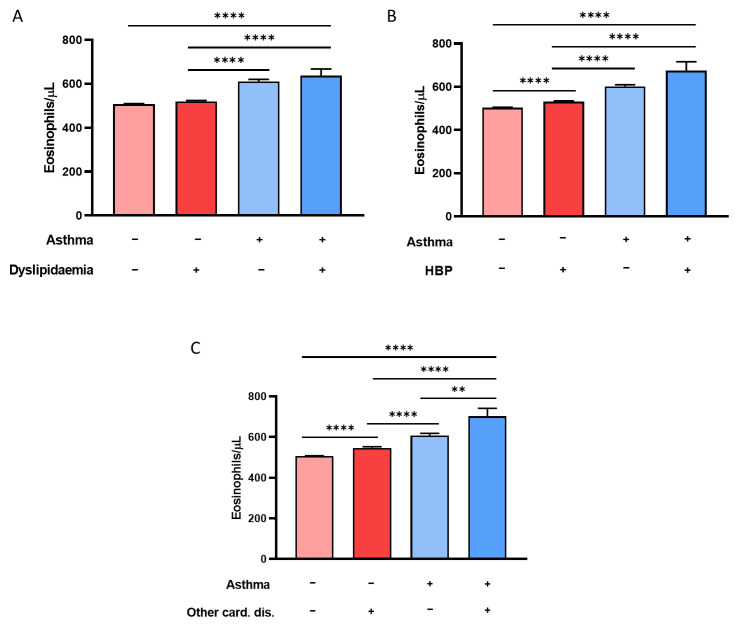
Cardiovascular diseases are associated with eosinophilia in asthmatic patients. (**A**) BEC in non-diseased subjects and subjects with dyslipidemia, with asthma, and with both. (**B**) BEC in non-diseased subjects, with HBP, with asthma, and with both. (**C**) BEC in non-diseased subjects (−/−) and in subjects with other cardiac pathologies, with asthma, and with both. ** = *p* < 0.005; **** = *p* < 0.0001. BEC: blood eosinophil count; HBP: high blood pressure. + sign represents presence of the disease and − sign represents absence of the disease.

With respect to metabolic disorders, we studied the influence of the mean BEC in asthmatic and nonasthmatic subjects over dyslipidemia (Figure 4A). Patients with both pathological conditions (640 ± 780 eosinophils/μL) developed significantly higher eosinophilia levels than non-diseased subjects (510 ± 500 eosinophils/μL; *p* < 0.0001) and patients with dyslipidemia (520 ± 430 eosinophils/μL; *p* < 0.0001), in spite of having values above 300 BEC/μL. The difference in BEC between asthmatics with dyslipidemia and those with asthma only was not statistically significant (610 ± 560 eosinophils/μL; *p* = 0.6).

Finally, with regard to cardiovascular risk, the subjects with the highest mean BEC were those diagnosed with asthma and hypertension (670 ± 1090 eosinophils/μL), as these individuals had significantly higher eosinophilia levels than subjects without asthma or HBP (500 ± 500 eosinophils/μL; *p* < 0.0001), hypertension (530 ± 470 eosinophils/μL; *p* < 0.0001), or asthma alone (600 ± 420 eosinophils/μL; *p* < 0.0001), as shown in Figure 4B. This same relationship was also observed when studying eosinophilia in patients with asthma and other cardiovascular diseases (Figure 4C). Asthmatic individuals developed significantly higher eosinophilia levels than subjects without cardiovascular diseases or with HBP alone, as seen in Figure 4A–C (*p* < 0.0001). It should be noted that, in all cases, non-diseased individuals had the lowest mean BEC.

### 3.4. Multiple Logistic Regression Models Show the Association between BEC Greater Than 600 Eosinophils/µL and Asthma, CRSwNP, COPD, and Hospitalization

Since we observed that blood eosinophilia equal to or greater than 600 eosinophils/μL significantly increased the association with asthma (Figure 2B), we assessed whether such a threshold value could also be useful as a potential biomarker for other diseases and comorbidities, using the OR for different respiratory, metabolic, and cardiovascular pathologies (Figure S2A). Regarding respiratory diseases, asthma (OR = 2.22; 95% CI, 2.10 to 2.14; *p* < 0.0001) and CRSwNP (OR = 2.11; 95% CI, 1.66 to 2.68; *p* < 0.0001) stood out. However, sleep apnea (OR = 1.16; 95% CI, 1.03 to 1.31; *p* = 0.02), COPD (OR = 1.38; 95% CI, 1.22 to 1.56; *p* < 0.0001), combined asthma and sleep apnea (OR = 1.81; 95% CI, 1.18 to 2.80; *p* = 0.009), and respiratory conditions other than those under study (OR = 1.33; 95% CI, 1.20 to 1.47; *p* < 0.0001) were also statistically significant. Moreover, studying metabolic and cardiovascular diseases, the OR was significant for dyslipidemia (OR = 1.10; 95% CI, 1.05 to 1.15; *p* = 0.0001), hypertension (OR = 1.14; 95% CI, 1.09 to 1.79; *p* < 0.0001), and other cardiovascular diseases (OR = 1.29; 95% CI, 1.21 to 1.37; *p* < 0.0001).

Next, we studied the association of diseases with asthma, independently of the mean BEC (Figure S2B). Only CRSwNP was significantly associated with asthma (OR = 3.40; 95% CI, 2.54 to 5.51; *p* < 0.0001). However, COPD (OR = 0.60; 95% CI, 0.46 to 0.78; *p* < 0.0001), other respiratory diseases (OR = 0.77; 95% CI, 0.63 to 0.94; *p* = 0.01), dyslipidemia (OR = 0.77; 95% CI, 0.70 to 0.84; *p* < 0.0001), hypertension (OR = 0. 69; 95% CI, 0.63 to 0.75; *p* < 0.0001), and other cardiovascular diseases (OR = 0.67; 95% CI, 0.59 to 0.75; *p* < 0.0001) showed a significant negative association with asthma. Nevertheless, these data were not adjusted for any other features, characteristics, or clinical data pertaining to this population.

**Figure 5:**
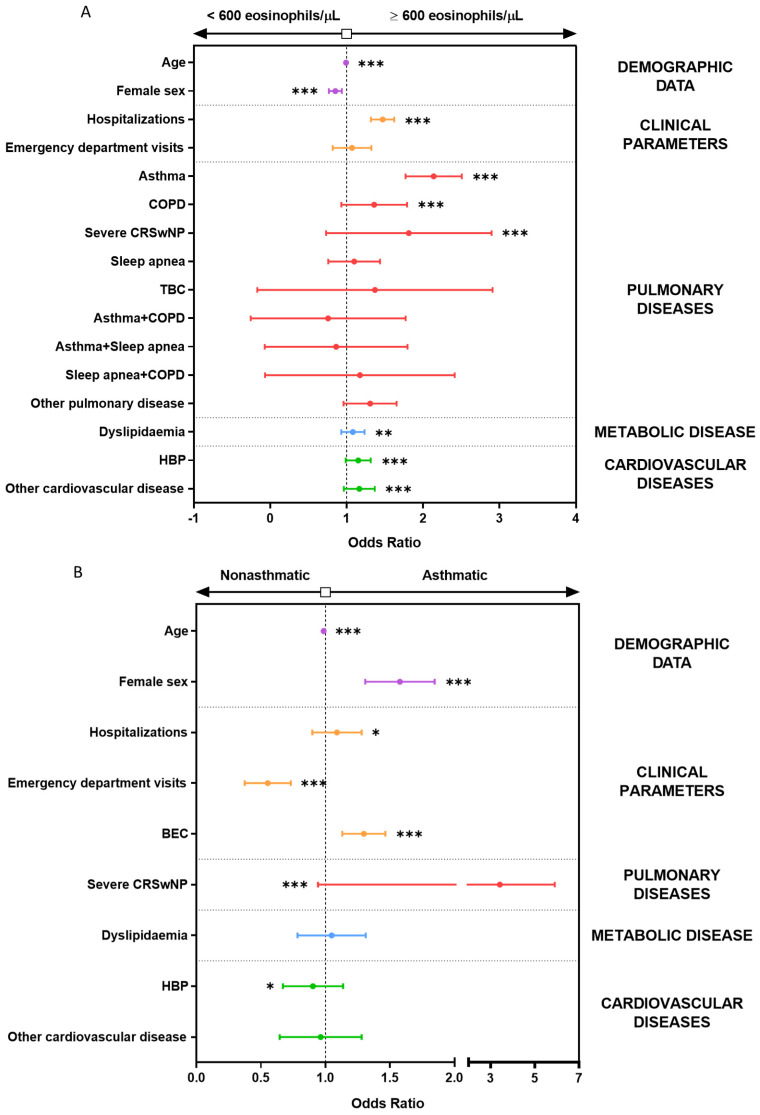
After adjustment for all predictive values between each other, BEC over 600 per microliter is associated with asthma, COPD, CRSwNP, and hospitalization, while asthma is related to BEC and CRSwNP. Odds ratio of respiratory, metabolic, and cardiovascular diseases according to (**A**) BEC or (**B**) asthma for the logistic regression models. * = *p* < 0.05; ** = *p* < 0.005; *** = *p* < 0.001. OR: odds ratio; CRSwNP: chronic rhinosinusitis with nasal polyps; COPD: chronic obstructive pulmonary disease; TBC: tuberculosis; HBP: high blood pressure; BEC: blood eosinophil count.

To account for interactions between the predictive variables of study, we performed logistic regression models. First, univariate logistic regression models were performed and values like emergency department visits with OR = 1.16 (95% CI, 1.06 to 1.28; *p* = 0.001) were not significant after multivariate correction with OR = 1.07 (95% CI, 0.97 to 1.17, *p* = 0.186; Supplementary Table S1). The same occurred for the relationship between the presence of asthma plus sleep apnea, with OR = 1.81 (95% CI, 1.16 to 2.79; *p* = 0.01) for the univariate and OR = 0.81 (95% IC, 0.51 to 1.26; *p* = 0.35) for multivariate model (Supplementary Table S1). As shown in Figure 5A, when multivariate logistic regression models were applied to all the variables studied, a BEC above 600 eosinophils/μL was associated with an increased frequency of hospitalization (OR = 1.47; 95% CI, 1.41 to 1.53; *p* < 0.001), CRSwNP (OR = 1.78; 95% CI, 1.39 to 2.26; *p* < 0.001), asthma (OR = 2.13; 95% CI, 1.99 to 2.29; *p* < 0.001), and COPD (OR = 1.35; 95% CI, 1.19 to 1.54; *p* < 0.001); the other variables were not associated with this degree of eosinophilia. Asthma was associated with female sex (OR = 1.57; 95% CI, 1.47 to 1.69; *p* < 0.001), CRSwNP (OR = 3.34; 95% CI, 2.47 to 4.46; *p* < 0.001), and blood eosinophilia (OR = 1.30; 95% CI, 1.23 to 1.36; *p* < 0.001), as depicted in Figure 5B. Hence, these models demonstrated that BEC, asthma, and CRSwNP were truly interdependent.

## 4. Discussion

In this study, we provide updated evidence on the importance of taking into account peripheral BEC levels in subjects with the presence of over three hundred blood eosinophils per microliter. Moderate eosinophilia at or above 600 eosinophils/µL was associated with an increased OR for asthma and COPD, a greater frequency of hospitalization, and a history of ESS in subjects with already the presence of over 300 BEC, which underscores the importance of considering blood eosinophil counts over 600 cells/µL, as a risk factor worth noting in subjects with current eosinophilia.

Historically, BEC cutoff values have been employed in clinical management, particularly in departments such as hematology, allergy, respiratory medicine, and tropical diseases, where this parameter is associated with specific diseases or conditions, such as asthma [40]. Furthermore, BEC is not considered a risk factor by itself; rather, it is evaluated in conjunction with other clinical data [41].

In light of this gap, we conducted an EMR-based retrospective study of subjects presenting blood eosinophilia as detected by blood tests over a two-year period to characterize these patients and determine their association with a different condition. Given the commonly used cutoff for severe asthma, one of the main diseases related to eosinophilia, we selected subjects with over 300 eosinophils/µL, as previous studies have demonstrated that at least one blood test result over this value is associated with a greater asthma exacerbation risk [21], and novel biological drugs that target eosinophils as benralizumab have this threshold as an optimal level for treatment selection and clinical improvement [42,43,44].

As our results confirm, eosinophilia tends to be higher among individuals with asthma, as previously described [45,46], and this pattern is independent of subjects’ age. In our study, the threshold most closely related to the presence of asthma is 600 eosinophils/µL, considering a population with already 300 blood eosinophils/μL. This finding contrasts with previous reports showing that the median blood eosinophil counts range from 157 to 280 cells/µL for asthma and 200 to 400 cells/µL for severe asthma, while controls and the general population have blood eosinophil counts of 100–160 cells/µL and 100–200 cells/µL, respectively [47].

Due to our study design, a significant percentage of our nonasthmatic population presented some diseases that could be related to this blood eosinophilia. This may alter the previously published ratios, considering factors such as hospitalization and other pathological conditions. When considering the 600 eosinophils/µL BEC level, asthma became significantly more frequent in our population, and the OR rose to 1.5 over this cutoff. For this reason, we suggest acknowledging the evaluation of a possible diagnosis of eosinophilic asthma for those subjects with BEC above 600 eosinophils/µL. Indeed, a high BEC could be important in defining patients with persistent eosinophilic asthma, and a high BEC from a single test (>470 eosinophils/µL) has been described as a predictor of this condition [48]. Moreover, BEC has been proposed as a biomarker for sputum eosinophilia in asthma, which proves the relation between blood and airway eosinophilia [49]. Earlier diagnosis would allow the prompt consideration of biologic drugs, which have been shown to significantly improve the quality of life in these patients [29,50].

When considering the frequency of respiratory diseases in our study, asthma was associated with the highest BEC, either alone or when cooccurring with other diseases. Interestingly, tuberculosis patients also had a high BEC, potentially related to disease pathogenesis [51] or treatment administration [52,53,54]. As for CRSwNP, numerous studies have reported a relationship between the presence of CRSwNP and the inflammatory role of eosinophils [55,56]. Asthmatic individuals with CRSwNP had significantly higher eosinophilia than patients with only one disease. Recently, Zhong et al. also described that BEC could be useful in distinguishing CRSwNP phenotypes and in predicting polyp recurrence [57]. Hence, it is of high importance to review BEC in routine clinical practice, especially in patients at greater risk of developing asthma and/or CRSwNP and in subjects with over 600 eosinophils/μL, due to an OR greater than 2 for both diseases. Indeed, current clinical practice guidelines consider BEC as a biomarker for both conditions [22,38].

Although COPD is generally not associated with high BEC, since the most common variant is neutrophilic [16], we found a relationship between BEC above 600 eosinophils/μL and COPD, and it was maintained in the multiple logistic regression model. Amini et al. also found a positive association between peripheral BEC and COPD in the Lifelines cohort from the Netherlands [58]. Other studies have also concluded that COPD patients have significantly elevated BEC [47,59]. However, it is important to mention that this positive association could be biased if the study subjects experience an exacerbation at the time of the blood test. Kim and Shawn defined high blood neutrophil or eosinophil counts as a necessary laboratory criterion for exacerbations in COPD [60]. Furthermore, the BEC may be useful in detecting COPD patients who stand to benefit from systemic corticosteroid treatment [61] and biologic therapies [28].

Subjects with metabolic and cardiovascular diseases had significantly higher BEC than subjects who were only asthmatic. Furthermore, an analysis of the OR for all of these diseases with respect to the degree of eosinophilia revealed a significant positive association with BEC higher than 600 eosinophils/μL. Based on these results, we hypothesized that a mild BEC is associated with dyslipidemia, hypertension, and other cardiovascular diseases. These preliminary results are consistent with those of Pongdee et al., who concluded that the prevalence of both diseases increases with a higher BEC [14]. However, when we ran a multinomial logistic regression model, this association was lost for dyslipidemia and reduced in the case of hypertension, although it remained significant. Even though the relationship between eosinophilia and metabolic syndrome has been described in numerous articles [17,18,47], Huang et al. demonstrated that the relationship was only true for neutrophils, monocytes, and basophils [62]. Regarding hypertension and other cardiovascular diseases, Hartl et al. found no association with BEC in the general population [8] and Amini et al. found no association between BEC and blood pressure [58]. Another consideration regarding eosinophilia in patients with hypertension may be the side effects of angiotensin-converting enzyme inhibitors (ACE inhibitors), one of the most common hypertension drugs [63]. These controversial results call for further research to clarify the relationship between BEC and metabolic and cardiovascular diseases.

Given the sound evidence pointing to a relationship between asthma and eosinophilia [16,46], we decided to study the association between asthma and the presence of respiratory, metabolic, and cardiovascular diseases. On individual analysis, we observed that being asthmatic is negatively associated with metabolic syndrome, HBP, and other cardiovascular diseases. However, as with eosinophilia, this relationship was lost on multiple logistic regression analysis, thereby only confirming the association with CRSwNP, as previously described [64]. It is noteworthy that although there was no significant association between hospitalization and asthma in this model, hospitalization did show a statistically significant association with BEC on multiple logistic regression, confirming that individuals with mild to severe eosinophilia are at a greater risk of needing medical attendance. This result could be linked not only to the eosinophils’ association with asthma, COPD, CRSwNP, and other comorbidities, but also to the possible presence of other eosinophil-derived pathologies, like or HES, EGPA, and, therefore, repeated blood counts must be addressed in order to uncover a persistent phenotype [4].

The main limitation of this research concerns the use of BEC as a constant parameter. Several studies have shown that BEC is mediated by multiple factors, such as diurnal variations, smoking, and exercise [47,65]. Herrett et al. explain that considering BEC as a dichotomous variable in clinical practice, with a single cutoff point, is overly simplistic [66]. Another limitation is that our study population is not representative of the entire population due to the selection of patients already presenting blood eosinophilia, conducted with the specific aim of characterizing this subgroup, which could imply that specific BEC cutoff values as disease biomarkers may vary for the general population. Moreover, only subjects who underwent a medical examination were included, and the study was conducted during the COVID-19 pandemic, which could have affected the BEC data [67].

## 5. Conclusions

These results emphasize the association of a high BEC with the presence of asthma, CRSwNP, increased hospitalization, and COPD. While specific BEC cutoff levels should be further confirmed in the general population, and further biomarker studies need to be performed to fully apply BEC levels as indicators for asthma comorbidities, augmented eosinophilia should be perceived as a relevant clinical indicator that can lead to a worse clinical trajectory, especially in those subjects with the presence of blood eosinophils over 300 per microliter.

## Figures and Tables

**Table 1:** Clinical characteristics of the study subjects.

	Total Population >300 BEC (n = 53,788)	Nonasthmatics >300 BEC (n = 49,909)	Asthmatics >300 BEC (n = 3879)	*p*-Value (Asthma vs. No Asthma)
Age (Mean ± SD)	48.5 ± 17.8	48.7 ± 17.8	45.5 ± 17.3	<0.0001
Female sex (n, %)	27,188 (50.5)	24,814 (49.7)	2374 (61.2)	<0.0001
BEC, eosinophils/μL (Mean ± SD)	510 ± 490	510 ± 480	610 ± 600	<0.0001
Hospitalizations (n, %)	20,824 (38.7)	19,278 (38.6)	1546 (39.9)	<0.0001
Emergency department visits (n, %)	51,218 (95.2)	47,626 (95.4)	3592 (92.6)	<0.0001
Cardiovascular diseases (n, %)				
HBP	11,755 (23.3)	11,076 (23.7)	679 (17.7)	<0.0001
Others	5266 (11.6)	4964 (11.9)	302 (8.3)	<0.0001
Metabolic diseases (n, %)				
Dyslipidemia	10,212 (20.2)	9574 (20.5)	638 (16.6)	<0.0001
Respiratory diseases (n, %)				
Asthma	3607 (6.7)	0 (0)	3879 (100)	<0.0001
Severe CRSwNP	280 (0.5)	222 (0.4)	58 (1.5)	<0.0001
Sleep apnea	1308 (2.4)	1308 (2.6)	84 (2.2)	ns
COPD	1214 (2.3)	1214 (2.4)	57 (1.5)	<0.0001
TBC	86 (0.2)	86 (0.2)	4 (0.1)	ns
Others	1710 (3.2)	1710 (3.4)	103 (2.7)	0.0100
Smoking habit (n, %)				
Smoker	11,951 (24.0)	11,196 (24.4)	755 (19.8)	<0.0001
Nonsmoker	28,985 (58.3)	26,653 (58.0)	2332 (61.2)	0.0002
Ex-smoker	8720 (17.5)	8003 (17.4)	717 (18.8)	0.0300

Note: BEC, blood eosinophil count; HBP, high blood pressure; CRSwNP, chronic rhinosinusitis with nasal polyps; COPD, chronic obstructive pulmonary disease; TBC, tuberculosis; ns: not statistically significant.

## Data Availability

The data that support the findings of this study are available from the corresponding author, V.d.P., upon reasonable request.

## Supplementary Materials

The following supporting information can be downloaded at: https://www.mdpi.com/article/10.3390/biom14010126/s1. Figure S1. Smokers have significantly lower mean BEC in both nonasthmatic and asthmatic subjects. BEC in asthmatic and nonasthmatic subjects according to their smoking habit. **** = *p* <0.0001; ** = *p* <0.005. BEC, Blood Eosinophil Count; Non-SM, Non-smoker; SM, Smoker; Ex-SM, Ex-Smoker. Figure S2. Asthma disease presents a significant association with CRSwNP. Odds ratio of respiratory, metabolic, and cardiovascular diseases according to (A), BEC or (B), asthma. * = *p* <0.05; ** = *p* <0.005; *** = *p* <0.001; **** = *p* <0.0001. OR: Odds Ratio; CRSwNP: Chronic Rhinosinusitis with Nasal Polyps; COPD: Chronic Obstructive Pulmonary Disease; TBC: Tuberculosis; HBP: High Blood Pressure; BEC: Blood Eosinophil Count. Table S1. Univariate and multivariate logistic regression model with BEC > 600 eosinophils/μL as the main variable. Table S2. Univariate and multivariate logistic regression model with asthma as the main variable.

## Abbreviations

ADCC	Antibody-dependent cellular cytotoxicity
AEs	Asthma exacerbations
BEC	Blood eosinophil count
COPD	Chronic obstructive pulmonary disease
CRSwNP	Chronic rhinosinusitis with nasal polyps
EGPA	Eosinophilic granulomatosis with polyangiitis
EMR	Electronic medical record
EoE	Eosinophilic esophagitis
ESS	Endoscopic sinus surgery
GEMA	Guidelines for Asthma Management
HBP	High blood pressure
HES	Hypereosinophilic syndrome
HU	Hospital Universitario
IL-5Rα	IL-5 alpha subunit receptor
MetS	Metabolic syndrome
OR	Odds ratio
SEA	Severe eosinophilic asthma
