# Supplementary material for: Moderate-High Blood Eosinophilia Is Associated with Increased Hospitalization and Other Asthma Comorbidities
Source: Biomolecules. 2024 Jan 18;14(1):126. doi: 10.3390/biom14010126 (PMC10813157; doi:10.3390/biom14010126)
Supplement: Supplementary file 1 [file biomolecules-14-00126-s001.zip › biomolecules-2756274-supplementary.pdf]

Supplementary material

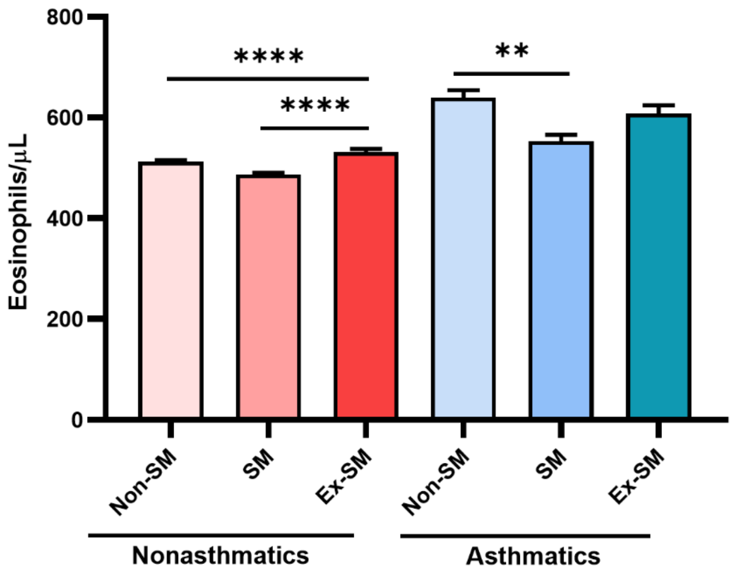

Figure S1. Smokers have significantly lower mean BEC in both nonasthmatic and asthmatic subjects. BEC in asthmatic and nonasthmatic subjects according to their smoking habit. ****= *P* <0.0001; **= *P* <0.005. BEC, Blood Eosinophil Count; Non-SM, Non-smoker; SM, Smoker; Ex-SM, Ex-Smoker.

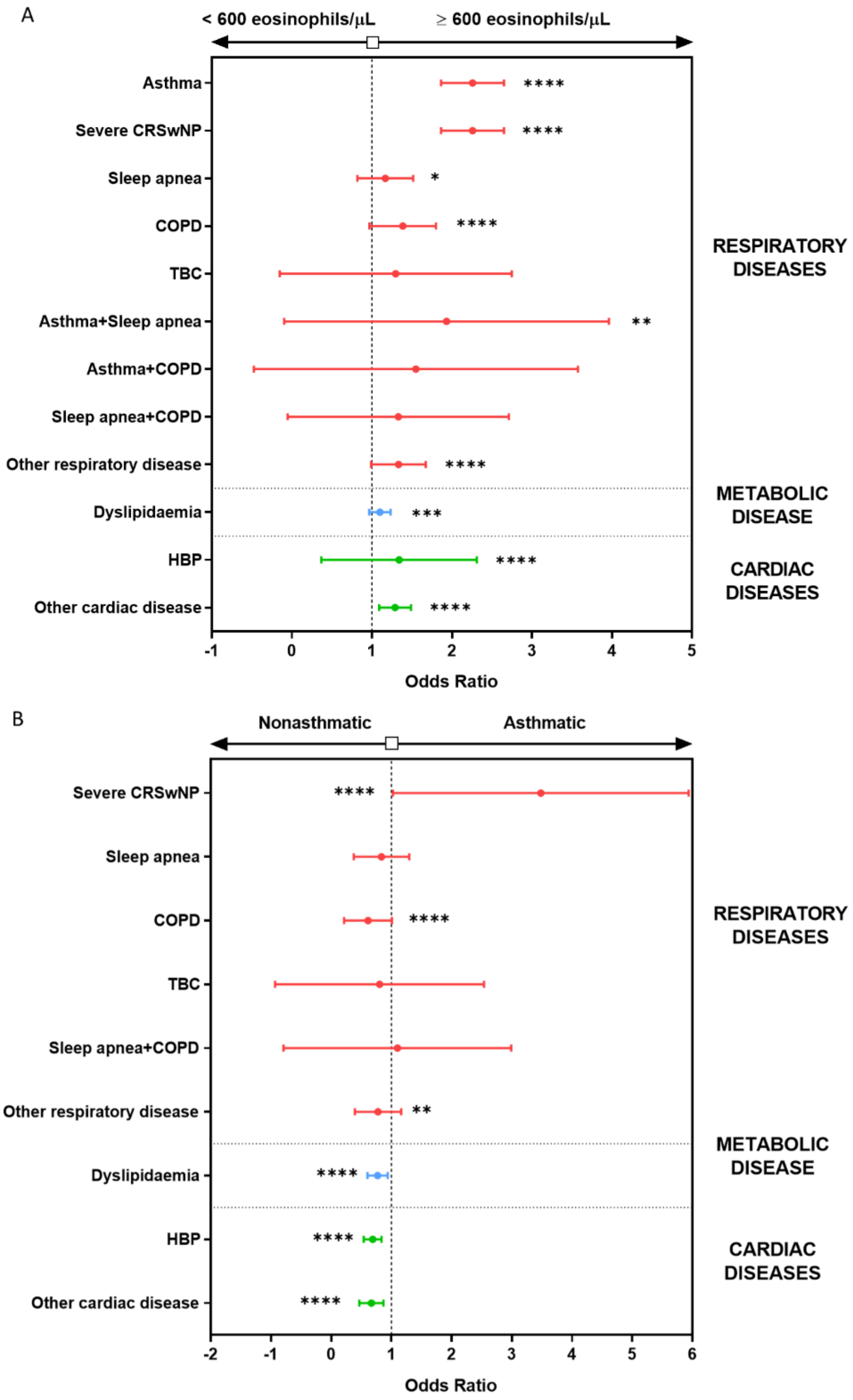

Figure S2. Asthma disease presents a significant association with CRSwNP. Odds ratio of respiratory, metabolic, and cardiovascular diseases according to (A), BEC or (B), asthma. * = P <0.05; ** = P <0.005; *** = P <0.001; **** = P <0.0001. OR: Odds Ratio; CRSwNP: Chronic Rhinosinusitis with Nasal Polyps; COPD: Chronic Obstructive Pulmonary Disease; TBC: Tuberculosis; HBP: High Blood Pressure; BEC: Blood Eosinophil Count.

Supplementary Table 1: Univariate and multivariate logistic regression model with BEC>600 eosinophils/µL as the main variable.

	UNIVARIATE		MULTIVARIATE	
**Variable**	**OR (95% CI)**	***P***	**OR (95% CI)**	***P***
**Age**	0.99 (0.99-0.99)	0.002	0.99 (0.99-0.99)	<0.001
**Female Sex**	0.87 (0.84-0.90)	<0.001	0.85 (0.82-0.89)	<0.001
**Hospitalizations**	**1.49 (1.43-1.54)**	**<0.001**	**1.47 (1.41-1.53)**	**<0.001**
**Emergency department visits**	1.16 (1.06-1.28)	0.001	1.07 (0.97-1.17)	0.186
**Severe CRSwNP**	**2.11 (1.66-2.67)**	**<0.001**	**1.78 (1.40-2.27)**	**<0.001**
**Dyslipidaemia**	1.12 (1.07-1.18)	<0.001	1.08 (1.02-1.14)	0.008
**HBP**	1.16 (1.11-1.21)	<0.001	1.15 (1.09-1.22)	<0.001
**Other cardiovascular disease**	**1.33 (1.25-1.41)**	**<0.001**	1.16 (1.08-1.25)	<0.001
**Asthma**	**2.10 (1.96-2.24)**	**<0.001**	**2.14 (1.99-2.29)**	**<0.001**
**Sleep apnea**	1.16 (1.03-1.311)	0.015	1.09 (0.96-1.24)	0.164
**COPD**	**1.38 (1.22-1.56)**	**<0.001**	**1.35 (1.19-1.54)**	**<0.001**
**TBC**	1.21 (0.75-1.90)	0.418	1.29 (0.80-2.03)	0.297
**Asthma + aleep apnea**	**1.81 (1.16-2.79)**	**0.010**	0.81 (0.51-1.26)	0.354
**Asthma + COPD**	**1.40 (0.79-2.40)**	**0.242**	0.69 (0.39-1.20)	0.189
**Sleep apnea + COPD**	1.25 (0.80-1.91)	0.312	1.11 (0.71-1.70)	0.642
**Other pulmonary disease**	**1.33 (1.20-1.47)**	**<0.001**	**1.30 (1.17-1.5)**	**<0.001**

Abbreviations: OR, Odds Ratio; 95% CI, 95% Confidence Interval; HBP, High Blood Pressure; CRSwNP, Chronic Rhinosinusitis with Nasal Polyps; COPD, Chronic Obstructive Pulmonary Disease; TBC, Tuberculosis; ns: not statistically significant.

Note: Values in bold are significant and with an OR lesser than 0.7 and/or higher than 1.3.

Supplementary Table 2: Univariate and multivariate logistic regression model with asthma as the main variable.

	UNIVARIATE		MULTIVARIATE	
**Variable**	**OR (95% CI)**	***P***	**OR (95% CI)**	***P***
**Age**	0.99 (0.99-0.99)	<0.001	0.99 (0.98-0.99)	<0.001
**Female sex**	**1.60 (1.49-1.71)**	**<0.001**	**1.58 (1.47-1.69)**	**<0.001**
**Hospitalizations**	1.05 (0.99-1.13)	0.131	1.09 (1.01-1.17)	0.021
**Emergency department visits**	**0.60 (0.53-0.68)**	**<0.001**	**0.55 (0.48-0.63)**	**<0.001**
**BEC**	1.29 (1.23-1.36)	<0.001	1.30 (1.23-1.37)	<0.001
**Severe CRSwNP**	**3.40 (2.52-4.51)**	**<0.001**	**3.35 (2.47-4.46)**	**<0.001**
**Dyslipidaemia**	0.83 (0.76-0.91)	<0.001	1.04 (0.94-1.16)	0.405
**HBP**	0.74 (0.68-0.81)	<0.001	0.90 (0.81-0.99)	0.048
**Other cardiovascular disease**	0.76 (0.68-0.86)	<0.001	0.96 (0.84-1.09)	0.524

Abbreviations: OR, Odds Ratio; 95% CI, 95% Confidence Interval; HBP, High Blood Pressure; CRSwNP, Chronic Rhinosinusitis with Nasal Polyps; COPD, Chronic Obstructive Pulmonary Disease; TBC, Tuberculosis; ns: not statistically significant.

Note: Values in bold are significant and with an OR lesser than 0.7 and/or higher than 1.3.
